# Establishment of a 24/7 robotic acute care surgery program at a large academic medical center

**DOI:** 10.1007/s00464-024-11036-x

**Published:** 2024-07-09

**Authors:** Daniel Gage, Taylor Neilson, Megan G. Pino, Daniel Eiferman, Jennifer Knight-Davis

**Affiliations:** 1 Department of General Surgery, The Ohio State University, 395 West 12th Avenue, Suite 662, Columbus, OH 43210 USA; 2 Medical Scientist Training Program, The Ohio State University College of Medicine, Columbus, OH USA; 3 Division of Trauma, Critical Care, and Burns, The Ohio State University, Columbus, OH USA

**Keywords:** Robotic surgery, Robotic surgery program, Acute care surgery, Emergency general surgery, Minimally invasive surgery, Laparoscopic surgery

## Abstract

**Background:**

For many years, robotic surgery has been an option for various elective surgical procedures. Though robotic surgery has not traditionally been the first choice for acute surgical patients, recent work has shown promise in broader applications. However, there are limited data regarding how to establish an institutional robotics program for higher acuity patients. This project aimed to map a pathway for the creation of an acute care surgery robotic program at a large academic medical center.

**Methods:**

Various stakeholders were gathered jointly with our surgical faculty: anesthesia, operating room leadership, surgical technologists, circulating nurses, Central Sterile Supply, and Intuitive Surgical Inc. representatives. Staff underwent robotics training, and surgical technologists were trained as bedside first assistants. Nontraditional robotic operating rooms were allocated for coordinated placement of appropriate cases, and pre-made case carts were arranged with staff to be available at all hours. A workflow was created between surgical faculty and staff to streamline add-on robotic cases to the daily schedule.

**Results:**

Six faculty and two fellows are now credentialed in robotics surgery, and additional surgeons are undergoing training. Numerous staff have completed training to perform operative assistant duties. The operating capacity of robotic acute care surgeries has more than doubled in just one year, from 77 to 172 cases between 2022 and 2023, respectively. Two add-on cases can be accommodated per day. Select patients are being offered robotic surgeries in the acute surgical setting, and ongoing efforts are being made to create guidelines for which patients would best benefit from robotic procedures.

**Conclusions:**

Launching a successful robotic surgery program requires a coordinated, multidisciplinary effort to ensure seamless integration into daily operations. Additional assistance from outside technology representatives can help to ensure comfort with procedures. Further studies are needed to determine the acute patient population that may benefit most from robotic surgery.

Minimally invasive surgery (MIS), comprised of laparoscopic surgery and robotic surgery, has been a validated, widely adopted alternative to open surgery for several decades. First reported in 1910, laparoscopic techniques were initially criticized and did not gain traction until much later in the century [1]. Today, approximately 15 million MIS procedures are performed annually, with the United States accounting for nearly 5 million cases [2]. These numbers continue to rise each year, creating a greater demand for MIS platforms, programs, and trained personnel.

MIS has profound benefits compared to open surgery, the most notable being reductions in operative and postoperative complications, hospital stay, recovery time, and postoperative pain [3]. MIS techniques have been implemented across most surgical subspecialties and have become the gold standard approach for the diagnosis and treatment of many thoracic and abdominal disorders. Some commonly performed MIS procedures are cholecystectomy, appendectomy, and hysterectomy [4–6]. Historically, these are more frequently performed in the ambulatory setting.

Within MIS, laparoscopic surgery and robotic surgery are comparable in terms of morbidity and mortality outcomes. Some studies have even reported lower total complication rates in patients undergoing robotic surgery, specifically for hysterectomy, gastrectomy, and rectal cancer resection procedures [7]. Though rates of conversion to open surgery and total postoperative length of stay are not significantly different between laparoscopic surgery and robotic surgery as a whole, several studies investigating specific surgical procedures have associated robotic surgery with lower conversion rates and shorter length of stay [8–13]. Higher postoperative cosmesis satisfaction, sexual function, and continence have been identified, indicating that robotic surgery may result in improved quality of life outcomes compared to laparoscopic surgery [8, 14–16]. Despite its potential for improved outcomes, robotic surgery has remained limited in clinical application due to higher healthcare system costs, longer total operative times, or physical space constraints.

Robotic surgery has been increasing in utility over the last decade, with an eightfold increase from 2012 to 2018 in the United States [17–19]. Further, robotic surgery has been gaining traction in the complex procedural setting due to the increased dexterity and precision it offers over traditional laparoscopic surgery. The most widely used robotic surgical system, the da Vinci Surgical System, utilizes specialty cameras that provide a three-dimensional view of the surgical field and endo-wristed instruments that provide tremor elimination [20, 21]. Surgeons and students can also train on the equipment, which acts as a virtual reality surgical simulator [22, 23]. Studies have shown that novice surgical trainees have a shorter learning curve for robotic compared to laparoscopic platforms, as well as better fatigue and physical comfort levels [24, 25]. As more qualified individuals become competent and confident in operating on robotic platforms, the number of robotic surgeries performed annually has increased, outcomes have improved, and interest in establishing robotic training programs at large academic medical centers has skyrocketed.

Recent work has begun examining outcomes of the use of robotic surgery in the acute surgical setting [26–28]. While its use may be contrary to historic trends for management of acutely ill surgical patients, robotic surgery has shown promise in this broader application. There is limited information, however, as to how to establish a program at a given institution [29]. In this study, we detail a pathway to the creation of a robotic program capable of completing acute care surgery cases at a large academic medical center.

## Methods

This single academic institution project evaluated a novel protocol for the integration of robotic acute care surgery into the division of Trauma, Critical Care, and Burns at the Ohio State University (OSU).

### Establishment of a multidisciplinary robotic surgery team

Various stakeholders involved in the operative department environment were identified. In addition to acute care surgeons, representatives from anesthesiology and Central Sterile Supply, operating room front desk staff, circulating nurses, surgical technologists, and Intuitive Surgical, Inc. staff were included. These individuals were mutually introduced to create an open channel of communication, led by a robotic surgical faculty liaison. Longitudinal, multidisciplinary discussions were held to identify barriers to the expansion of robotic acute care surgery and to workshop possible interventions.

### Surgical training in robotics

Surgeons obtained institutional certification to complete robotics cases through completion of their residency training or through an apprenticeship program. The apprenticeship program was led by experienced robotic surgeons and run collaboratively between the Division of Trauma, Critical Care, and Burns and the Division of General and Gastrointestinal Surgery, Minimally Invasive Surgery. Existing surgical faculty were eligible to obtain their robotics certification after successful completion of five robotics cases as an apprentice. A training program was developed specifically for operating room staff, such as surgical technologists and circulating nurses, to increase familiarity with robotics cases. The program included lectures and workshops led by a representative from Intuitive Surgical, Inc. This provided staff with additional exposure to robotic surgery, both in and out of the operating room.

### Robotic surgery platform

OSU utilizes the da Vinci Surgical System by Intuitive Surgical, Inc. for all robotic surgeries.

### Allocation of robotically capable operating rooms and scheduling of robotic surgery cases

At our institution, there are four designated operating rooms capable of accommodating robotic platforms on any given day. Surgeons who identified a need for a robotics case worked in coordination with the robotics scheduling team to book surgical cases in robotically capable operating rooms. The operating room front desk and robot program coordinators were contacted to assess availability of such operating rooms. Whenever possible, cases were scheduled on an elective basis. When cases were deemed emergent, additional scheduling considerations were given and changes to existing cases were occasionally made to accommodate the new robotics cases.

### Preparation of surgical case carts

Upon scheduling of robotic surgical cases, Central Sterile Supply was contacted and notified of the need for sterile robotic surgery case carts to be prepared. Designated universal da Vinci Surgical System robotic case carts contained the following instruments: Monopolar curved scissors, Maryland Dissector, Tip Up Grasper, Large Needle Driver, Cadiere Grasper, and Prograsp. For cases involving cholecystectomy, an additional two large clip appliers were added. Two universal case carts were prepared in advance to allow for add-on robotic surgery cases each day.

### Pre- and intraoperative workflow

**Fig. 1:**
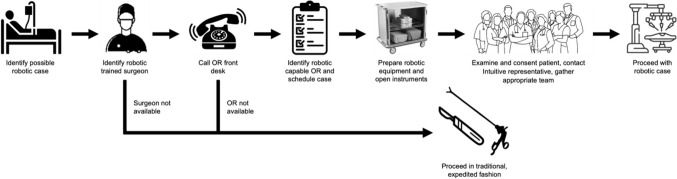
Workflow for robotic acute care surgery cases at our institution. These are the steps required to identify both a case and surgeon so they can be added onto the operating room schedule each day

Circulating nurses and surgical technicians prepared robotic and open instruments in the standard, sterile fashion. Anesthesia and surgery preoperative evaluations and consents were obtained from patients in standard fashion. An on-site Intuitive Surgery, Inc. representative was consulted for all robotic cases and was available to assist with operating room setup, technical support, and troubleshooting. During cases, Central Sterile Supply was available to provide additional sterile instruments, as needed. Surgical technologists performed bedside assistant duties. If needed, surgical residents also functioned as bedside assistants. This workflow is outlined in Fig. 1.

## Results

The Ohio State University (OSU) is a large academic medical center containing 1,404 beds. The Division of Trauma, Critical Care, and Burns currently employs 15 full-time faculty and two fellows who complete cases independently. Annually, the division completes over two thousand cases at OSU Main Campus and a smaller community hospital in Columbus, Ohio.

OSU was among the first academic institutions to adopt the da Vinci Surgical System. In 2023, 3,485 robotic cases were completed at OSU, spanning multiple specialties, including general surgery, colorectal surgery, surgical oncology, urology, and obstetrics and gynecology.

The implementation of OSU’s robotic surgery program led to the formal training of dozens of individuals at various levels of employment. Within the Division of Trauma, Critical Care, and Burns, six acute care surgeons are now certified to complete robotic surgeries independently. All operating room staff have completed additional in-service training and are now able to skillfully aid these surgeons during robotic procedures.

As the program has expanded, the division has obtained block time to place appropriate acute and elective outpatient cases. This required a multidisciplinary effort between surgeons and operating room staff to coordinate new robotic procedures with minimal disruption to daily operating room flow. Furthermore, the program and clarified workflow allowed for accommodation of robotic cases overnight and on non-business days, leading to 24/7 availability for patients.

**Fig. 2:**
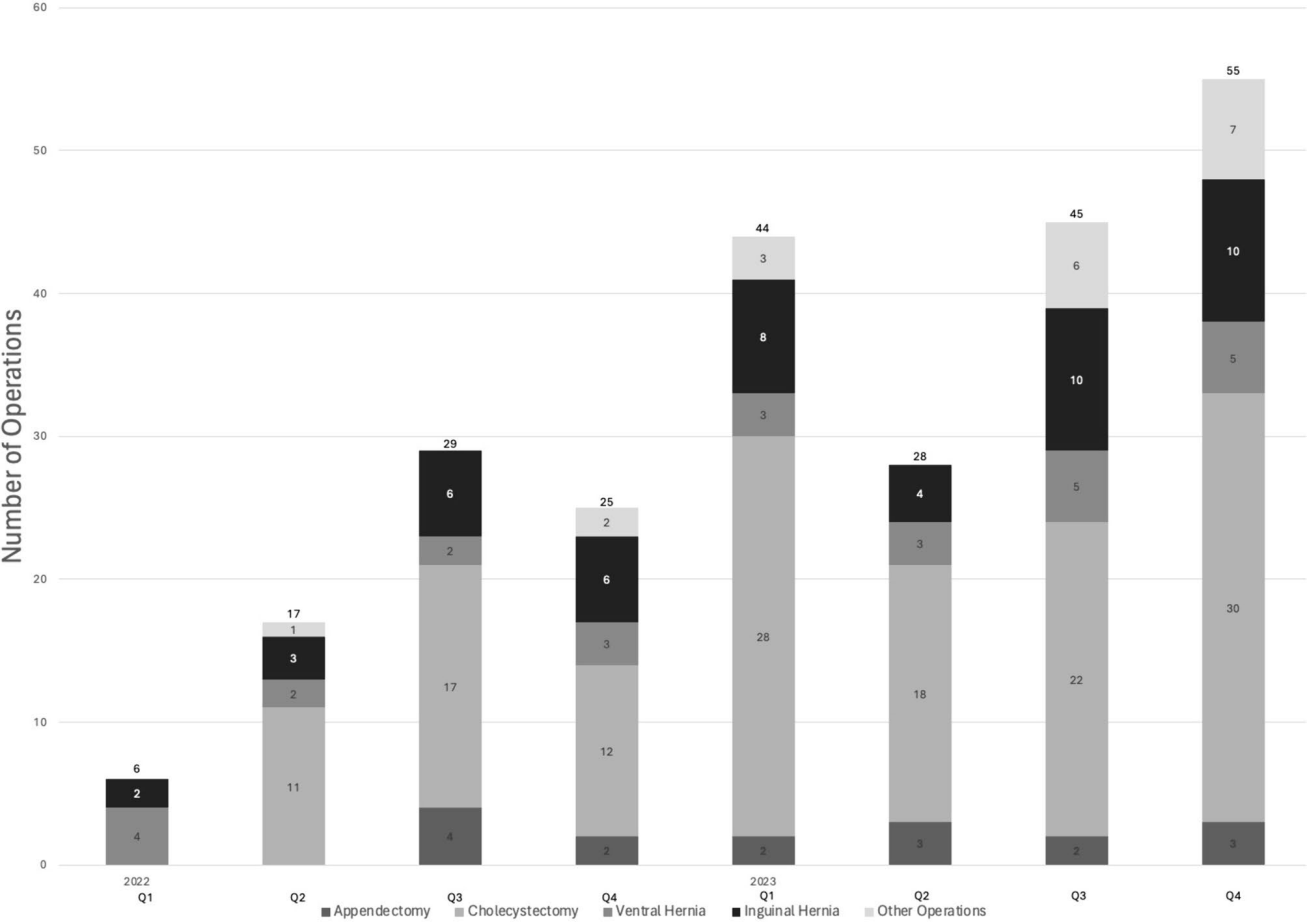
Increase in robotic acute care surgery cases performed at OSU from 2022 to 2023. Since robotic acute care surgical cases began being performed at OSU in early 2022, the number of robotic cases completed has steadily increased. Data are stratified by quarterly totals per calendar year (2022, *n* = 77; 2023, *n* = 172)

Since the formal initiation of robotic acute care surgery cases at OSU in early 2022, there has been a gradual increase in the number of cases completed within the division. A total of 77 robotic cases were completed in 2022, and a total of 172 robotic cases were completed in 2023, a 223% increase. The majority of cases completed included cholecystectomy, inguinal hernia repair, ventral hernia repair, and appendectomy (Fig. 2). These numbers continue to rise as more trained individuals, robotic equipment, and robotically capable operating rooms become available.

## Discussion

Robotic surgery has long been an option for patients undergoing various elective surgical procedures. Multiple studies have offered comparative analysis between traditional laparoscopic techniques and newer robotic ones, and recent work has begun examining outcomes of the use of robotic surgery in the acute surgical setting [4, 30, 31]. Patient safety is of upmost importance in the consideration of adoption of new surgical techniques and modalities. Data have suggested similar outcomes in robotic surgery compared to more traditional approaches [32–34], and some larger medical centers have begun developing programs to offer these interventions to their patients [28].

With advancements in minimally invasive surgical techniques, increasingly more procedures are being performed using robotic platforms. Thus, the ability to operate robotically is becoming a vital skillset required of all surgeons. Acute care surgeons are often urgently consulted intraoperatively to evaluate acute pathologies and intervene rapidly. Without surgeon knowledge of robotic systems, patients may be subjected to more invasive procedures, leading to increased morbidity, lengths of stay, and recovery times. Exposure to robotic surgery has become a part of fellowship training in other programs across the country [35] and remains a core component of OSU’s curriculum at both the residency and fellowship levels. Our surgical residents and fellows routinely participate in robotic cases across all years of their training, underscoring the importance of such training in the foundational development of surgeons.

Though robotic surgery has been emerging as a modality for the management of acutely ill patients, the availability of robotic platforms on short notice remains challenging during typical business hours. Scheduled robotics cases often utilize available consoles during working daytime hours – hours which acute care cases do not necessarily conform to. Our experience at OSU has demonstrated the importance of multidisciplinary collaborations to make additional staff and resources available for robotics cases to proceed at any hour. Previous work has demonstrated that robotic procedures like cholecystectomy that occur outside of typical business hours are equally as safe as those that occur during the day [36].

Engaging all participatory parties in the development of OSU’s robotics program was a key component to its early success. Prior to the program’s development, robotic surgery cases were solely performed on an elective basis at OSU. This meant, previously, operating room staff had at least one day to prepare for cases. With the introduction of daily add-on robotics cases, the streamlined, advance preparation of universal robotic case carts by Central Sterile Supply staff members was essential to feasibly integrate additional robotics cases into daily operations. Collaboration between faculty and operating room staff led to the creation of a standardized acute care surgery set that could be applied to most cases completed within the Division of Trauma, Critical Care, and Burns.

Surgical robotics training for not only surgeons but also operating room staff was another crucial component of the program. OSU surgical residents and fellows were provided ample opportunities to practice on robotic platforms through virtual reality simulations and to hone their skills in the operating room during live cases. Current surgical faculty were offered robotics training through an apprenticeship model, derived through a close partnership with colleagues specializing in elective minimally invasive surgery. As surgeons completed cases on an elective basis and gained the confidence and competence necessary to complete cases independently, they graduated from apprentices and became teachers to new apprentices. Interested operating room staff were provided formal training in robotic assistant duties, both by surgical faculty and by the Intuitive Surgical, Inc. Representative. After those who were interested were trained, additional efforts have continued, and all staff are now trained to participate in these cases.

This peer-inclusive, growth-oriented model allowed for a gradual expansion of individuals at OSU with surgical robotics expertise. This subsequently increased the availability of trained individuals and the possibility of completing a robotics procedure at any given time. At first, our institution underwent a three-month trial period where cases were carefully selected with interested staff members as well as our Intuitive, Inc. representative, who all volunteered to be available as a call team when a potential case was identified. This allowed for the early identification of any possible pitfalls which could be addressed before additional expansion. Now, multiple surgical teams can be assembled around the clock for emergent cases, limited only by the availability of a robotic platform and robotically capable operating room. Resources can be quickly organized for emergent add-on robotics cases in the same way they are coordinated for other modalities. Again, we emphasize the importance of a coordinated effort by all invested team members to successfully scale up the team’s robotic proficiency and capacity for additional cases. As a continued effort to provide quality care in this setting, our surgeons and operating room staff work in conjunction to evaluate all cases completed robotically with special attention to cases completed outside of regular business hours to ensure the appropriateness of case selection and utilization of resources during this time. There are limitations to the generalizability of this study as it was performed at a single, large academic medical center. Additionally, we did not distinguish between elective verses non-elective operative cases in our initial assessment of the ability to scale our program.

In conclusion, robotic surgery is a validated, safe method for surgical intervention of many acute care pathologies, and its applications should continue to be expanded. Through implementation of robotic surgery programs such as the one outlined in this study, medical centers may be able to complete both outpatient and emergent robotic surgery cases on a 24-h basis. Collaborating openly with all stakeholders in the robotic operating room environment and working to address unique barriers to completion of safe, standardized robotic cases at each institution will be key to individual programs’ success. Additional studies from our group will seek to compare outcomes from the use of robotics in acute care surgery to more traditional modalities of acute care surgery. Special attention will be given to comparison of cases completed electively through our group as well as add-on or after-hours cases. We hope to use these data to determine which patients may benefit most from robotic surgery intervention as well as how our robotics program can address disparities for patients undergoing acute care surgery.
